# Supplementary material for: Novel Driver Strength Index highlights important cancer genes in TCGA PanCanAtlas patients
Source: PeerJ. 2022 Aug 11;10:e13860. doi: 10.7717/peerj.13860 (PMC9375969; doi:10.7717/peerj.13860)
Supplement: Supplemental Information 2 [file peerj-10-13860-s002.zip › COHORTS/genes plots/cohorts/2021_8_16_14_9_distribution_cohorts_KIRC.pdf]

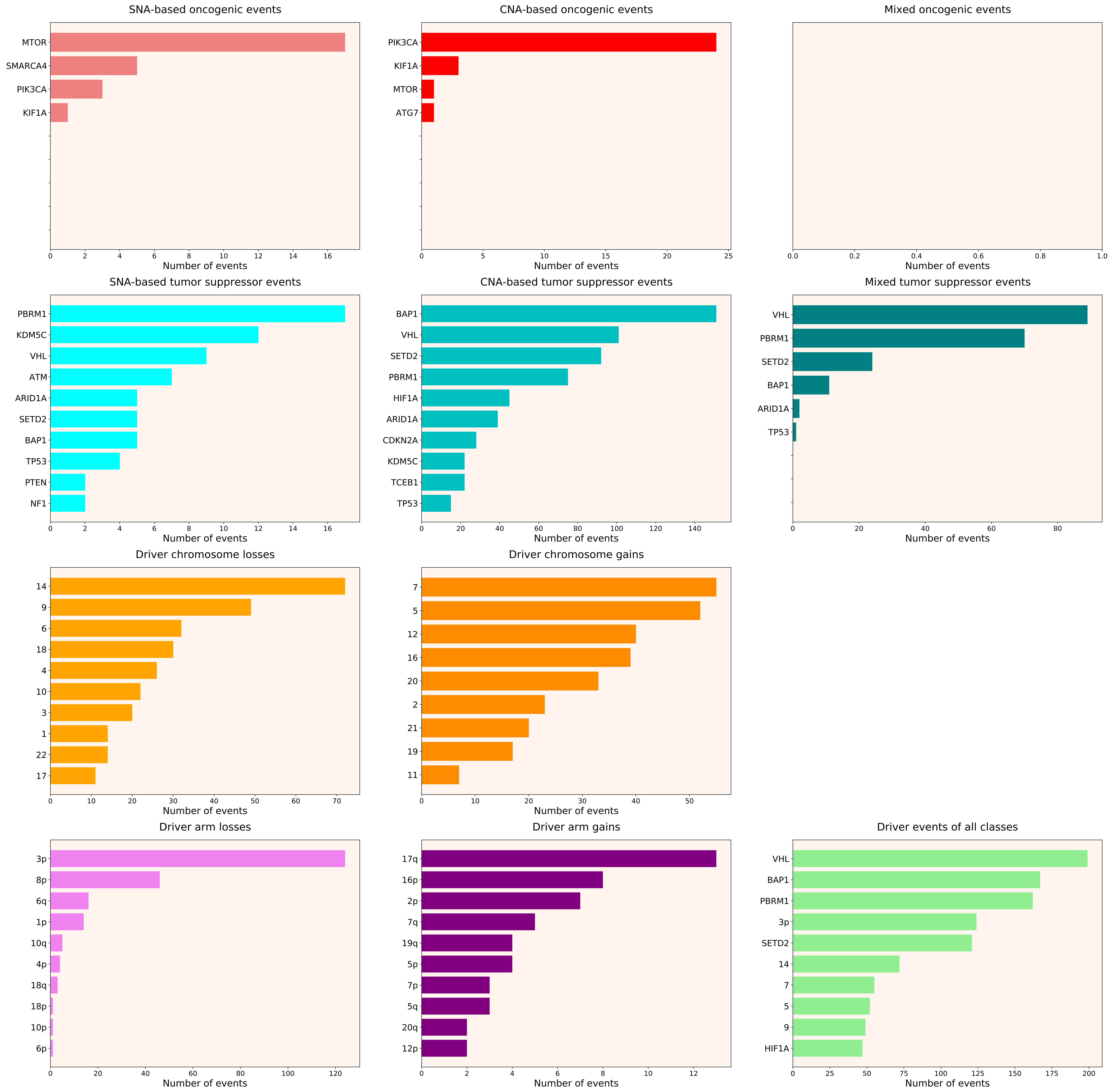

SNA-based oncogenic events
MTOR
SMARCA4
PIK3CA
KIF1A
Number of events
CNA-based oncogenic events
PIK3CA
KIF1A
MTOR
ATG7
Number of events
Mixed oncogenic events
Number of events
SNA-based tumor suppressor events
PBRM1
KDM5C
VHL
ATM
ARID1A
SETD2
BAP1
TP53
PTEN
NF1
Number of events
CNA-based tumor suppressor events
BAP1
VHL
SETD2
PBRM1
HIF1A
ARID1A
CDKN2A
KDM5C
TCEB1
TP53
Number of events
Mixed tumor suppressor events
VHL
PBRM1
SETD2
BAP1
ARID1A
TP53
Number of events
Driver chromosome losses
14
9
6
18
4
10
3
1
22
17
Number of events
Driver chromosome gains
7
5
12
16
20
2
21
19
11
Number of events
Driver arm losses
3p
8p
6q
1p
10q
4p
18q
18p
10p
6p
Number of events
Driver arm gains
17q
16p
2p
7q
19q
5p
7p
5q
20q
12p
Number of events
Driver events of all classes
VHL
BAP1
PBRM1
3p
SETD2
14
7
5
9
HIF1A
Number of events
